# Indoor Cycling Energy Expenditure: Does Sequence Matter?

**DOI:** 10.3390/ijerph18030870

**Published:** 2021-01-20

**Authors:** Cristina Cortis, Andrea Fusco, Mitchell Cook, Scott T. Doberstein, Cordial Gillette, John P. Porcari, Carl Foster

**Affiliations:** 1 Department of Human Sciences, Society and Health, University of Cassino and Lazio Meridionale, 03043 Cassino, Italy; andrea.fusco@unicas.it; 2 Department of Exercise and Sport Science, University of Wisconsin-La Crosse, La Crosse, WI 54601, USA; cookmp262@gmail.com (M.C.); sdoberstein@uwlax.edu (S.T.D.); cgillette@uwlax.edu (C.G.); jporcari@uwlax.edu (J.P.P.); cfoster@uwlax.edu (C.F.)

**Keywords:** physiological markers, perceptual responses, sRPE, high intensity interval training, intensity sequencing

## Abstract

Although cycling class intensity can be modified by changing interval intensity sequencing, it has not been established whether the intensity order can alter physiological and perceptual responses. Therefore, this study aimed to determine the effects of interval intensity sequencing on energy expenditure (EE), physiological markers, and perceptual responses during indoor cycling. Healthy volunteers (10 males = 20.0 ± 0.8years; 8 females = 21.3 ± 2.7years) completed three randomly ordered interval bouts (mixed pyramid—MP, ascending intervals—AI, descending intervals—DI) including three 3-min work bouts at 50%, 75%, and 100% of peak power output (PPO) and three 3-min recovery periods at 25% PPO. Heart rate (HR) and oxygen consumption (VO_2_) were expressed as percentages of maximal HR (%HR_max_) and VO_2_ (%VO_2max_). EE was computed for both the work bout and for the 5-min recovery period. Session Rating of Perceived Exertion (sRPE) and Exercise Enjoyment Scale (EES) were recorded. No differences emerged for % HR_max_ (MP = 73.3 ± 6.1%; AI = 72.1 ± 4.9%; DI = 71.8 ± 4.5%), % VO_2max_ (MP = 51.8 ± 4.6%; AI = 51.4 ± 3.9%; DI = 51.3 ± 4.5%), EE (MP = 277.5 ± 39.9 kcal; AI = 275.8 ± 39.4 kcal; DI = 274.9 ± 42.1 kcal), EES (MP = 4.9 ± 1.0; AI = 5.3 ± 1.1; DI = 4.9 ± 0.9), and sRPE (MP = 4.9 ± 1.0; AI = 5.3 ± 1.1; DI = 4.9 ± 0.9). EE during recovery was significantly (*p* < 0.005) lower after DI (11.9 ± 3.2 kcal) with respect to MP (13.2 ± 2.5 kcal) and AI (13.3 ± 2.5 kcal). Although lower EE was observed during recovery in DI, interval intensity sequencing does not affect overall EE, physiological markers, and perceptual responses.

## 1. Introduction

Although the benefits of regular exercise are widely accepted, 1 in 4 adults and more than 80% of adolescents are not active enough [1], with “lack of time” reported to be the biggest barrier to regular exercise [2]. Therefore, time efficient exercise routines are seen as desirable solutions to increase exercise participation. In particular, high intensity interval training (HIIT) has been shown to elicit similar or superior performance improvements and physiological adaptations as steady state training, with a significantly shorter time commitment [3].

Tabata et al. [4] demonstrated that HIIT protocols increased both aerobic and anaerobic capacities, whereas moderate intensity steady state aerobic training only produced improvements in aerobic capacity. This dual training effect influences performance, as both energy systems are used to varying degrees in all events, and improved anaerobic capacity is useful at the beginning and at the end of various events [5]. Additionally, Tabata et al. [6] found that work to rest ratios influence the stress load experienced by aerobic and anaerobic mechanisms. Extended recovery periods prevented either pathway from reaching maximal workloads. In accordance with this, Gosselin et al. [7] showed higher work to rest ratios elicited higher oxygen consumption (VO_2_), heart rate (HR), Rating of Perceived Exertion (RPE), and blood lactate concentration ([HLa]), but lower total energy expenditure (EE) compared to 1:1 ratios and steady state exercise. These findings suggest that the choice of work to rest ratios during HIIT depends on the desired physiological and perceptual responses.

The implementation of interval training as an alternative for steady state aerobic exercise must be viewed within the context of pacing strategies utilized within aerobic events. Foster et al. [8] defined pacing as the regulation of EE to complete a task in as little time as possible, while minimizing homeostatic disturbances. The requirements of race type and duration play a role in determining which strategy is the most effective [5,8,9,10]. Early use of high power, and uneven distributions of effort within an event, increase physiological and perceptual responses while decreasing performance capacity [11,12,13]. These studies suggest that variation of intensity within an aerobic exercise bout can affect overall performance, physiological responses, and perceptual feedback. However, how these same findings from single events may translate to HIIT, performed as a fitness exercise, as opposed to aerobically-based performance events (e.g., time trial competition), needs to be determined. Kang and colleagues [14] evaluated the effects of different mixed intensity order (ascending vs. descending) on cardiorespiratory, metabolic, and perceptual responses in apparently healthy adults. Results showed elevated VO_2_ and HR during the lower intensity workloads and decreased RPE responses during the higher intensity workloads of a descending protocol, compared to an ascending protocol. However, no EE differences were reported. These findings suggest that selecting higher intensities early during a workout session is associated with more favorable RPE, while generating similar EE.

Instructor-led cycling classes (i.e., indoor cycling) are a popular way to integrate HIIT in a group setting. Indoor cycling tends to combine aspects from aerobic training, interval training, and mixed intensity training, and may lead to very high within-workout values for VO_2_, HR, and [HLa] [15,16]. During a class, intensity is modified by changing interval intensity level, sequencing, and work to recovery ratios. However, it has not been established whether intensity order can alter physiological and perceptual responses within a workout. As developing a class structure that is both effective and enjoyable can help promote exercise participation, the aim of this study was to determine the effects of interval intensity sequencing on energy expenditure, physiological markers, and perceptual responses during indoor cycling.

## 2. Materials and Methods

Eighteen college-age and recreationally active (exercising >3 days per week for at least 30 min per session) subjects (males n = 10, females n = 8) provided written informed consent to participate in this study, which was approved by the Institutional Review Board for the Protection of Human Subjects at the University of Wisconsin-La Crosse (approval number: 45CFR46.46.110; date: 8 September 2016). Subjects were screened using the Physical Activity Readiness Questionnaire (PAR-Q) and the American Heart Association Health/Fitness Pre-Participation Screening forms to identify individuals with contraindications to participation [17].

All subjects completed four experimental sessions on an electronically braked cycle ergometer (Lode Excalibur, Groningen, Netherlands), which were organized within a 14-day period, with at least 48 h between sessions. Subjects were tested >3-h postprandial, had refrained from alcoholic consumption and heavy exercise > 24 h prior to testing, and abstained from caffeine consumption >6 h prior to testing. During the first session, subjects performed an incremental maximal exertion test to determine peak power output (PPO), maximal VO_2_ (VO_2max_) and maximal HR (HR_max_). The incremental test protocol began at 25 W and increased by 25 W every 2 min, until volitional fatigue. PPO was recorded at the end of the test based on the highest completed stage and the proportional time during incomplete stages.

Participants’ descriptive statistics are reported in Table 1.

**Figure 1:**
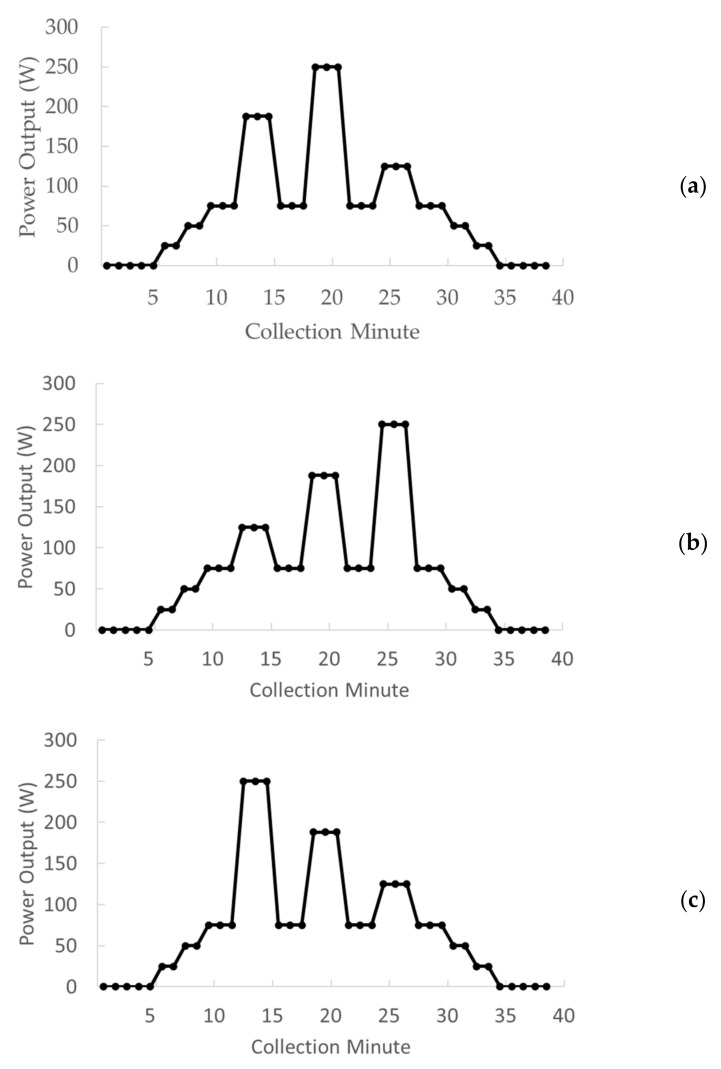
Schematic representation of the mixed pyramid (**a**), ascending (**b**), and descending (**c**) interval indoor cycling sessions. The data are presented for a subject with a peak power output of 250 W, although in the protocol, the workloads were adjusted to the individual peak power output.

Subsequently, subjects performed three randomly ordered interval bouts: mixed pyramid (MP), ascending (AI), and descending (DI) intervals, including three 3-min work bouts at workloads corresponding to 50%, 75%, and 100% of PPO and three 3-min recovery periods at 25% PPO following each interval periods. Each session started with a 5-min baseline period at rest followed by a 7-min warm-up including 2 min at 25 W, 2 min at 50 W, and 3 min at 75 W. All sessions concluded with a 4-min cool-down, mirroring the first two stages of the warm-up (50 W and 25 W) and a 5-min post-exercise resting period, performed sitting on the ergometer. The exercise portion of each session lasted 18 min. During MP, intervals were ordered as 75%, 100%, 50% of the individual PPO, during AI as 50%, 75%, 100% of the individual PPO, and during DI as 100%, 75%, 50% of the individual PPO. The mean % of PPO for all sessions was the same (41.7 ± 2.7). A schematic representation of the power output during the three experimental session is shown in Figure 1.

Subjects were instructed to maintain a pedaling rate of ~90 revolutions per minute throughout each session. Respiratory gas exchange was measured using a mixing chamber based open-circuit spirometry system (AEI Moxus, Pittsburg, PA, USA). Before each experimental session, known gas mixtures (16% O_2_, 4% CO_2_) and room air were used to calibrate the gas analyzer. Expiratory volume calibration was completed using a 3L syringe.

HR and VO_2_ were measured and expressed as percentages of HR_max_ (%HR_max_) and VO_2max_ (%VO_2max_). EE was considered for both the work bout and for the 5-min recovery period. Each liter of VO_2_ was assumed to yield 5 kcal·min^−1^.

Since recent evidence suggests that RPE scales are interchangeable [18,19], in the present study the Category Ratio 10 scale was used to monitor exercise intensity. Standardized instructions were provided prior to each session [20] and session Rating of Perceived Exertion (sRPE) was collected ~30 min after the end of the session to ensure that the perceived exertion referred to the whole session rather than to the most recent exercise intensity [21]. The Exercise Enjoyment Scale (EES) was administered at the end of each session to evaluate the individuals’ enjoyment [22].

### Statistical Analysis

Normal distribution was verified by the Shapiro-Wilk test and means and standard deviations were calculated for all variables. Statistical significance was set at *p* < 0.05. One-way analysis of variance (ANOVA) for repeated measures was applied to % HR_max_, % VO_2max_, sRPE, EE during the work bout and for the 5-min recovery period. If the overall F test was significant, post-hoc Tukey comparisons were used. The Stata statistical software version 14.1 (StataCorp, College Station, TX, USA) was used for statistical analysis.

## 3. Results

**Figure 2:**
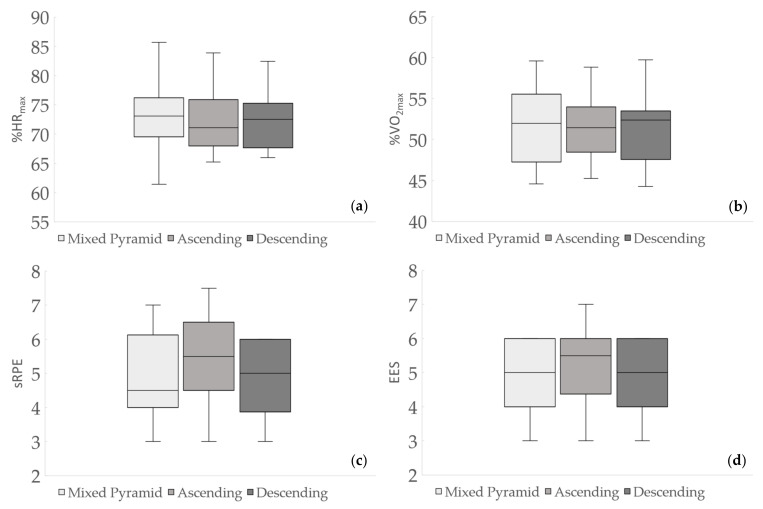
Percentages of maximal heart rate (% HR_max_), (**a**), oxygen consumption (% VO_2max_), (**b**), session Rating of Perceived Exertion (sRPE), (**c**), and Exercise Enjoyment Scale (EES), (**d**) responses of mixed pyramid, ascending and descending intervals during indoor cycling sessions.

**Figure 3:**
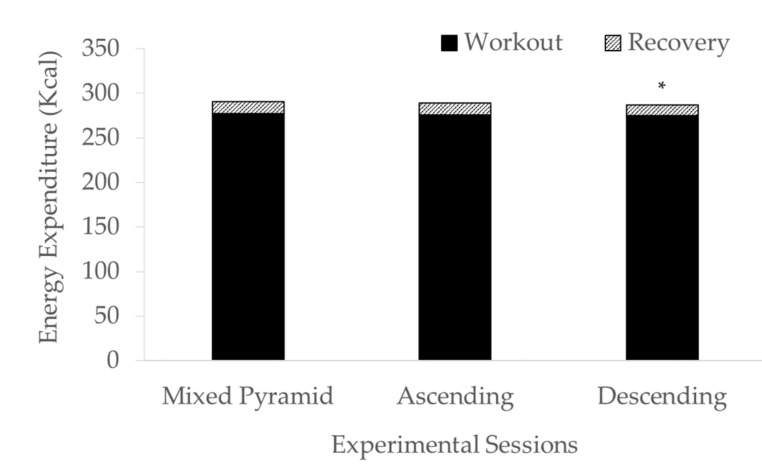
Energy expenditure during the workout and the recovery phase of the indoor cycling sessions. * denotes significantly (*p* < 0.005) lower energy expenditure during recovery than workout in descending intervals with respect to mixed pyramid and ascending intervals.

No significant differences were found for % HR_max_, % VO_2max_, sRPE, EES (Figure 2) and EE during the work bout. Differences (*p* < 0.005) emerged only for EE during the recovery period following DI (Figure 3).

## 4. Discussion

The main finding of this study was that although lower EE was observed during the recovery period in DI, interval intensity sequencing did not affect overall EE, physiological markers, and perceptual responses during cycling, suggesting that group cycling instructors can vary workout structure to promote adherence and maintain enjoyability, while achieving the same EE.

Total EE in MP, AI, and DI protocols did not show any difference, supporting findings from previous research in which total EE was not affected by intensity order [14]. Since interval-based exercise, such as the one proposed in indoor cycling classes, can provide a time-efficient exercise session [3], instructors are suggested to administer exercise sessions of mixed intensities, with comparable total workload, while keeping the same energetic demand. This will allow exercise sessions to be differentiated based on the intensity sequencing providing various exercise session experiences to promote adherence and avoid boredom within a given workout routine. Moreover, since an EE of at least 300 kcal per session is recommended to maintain a healthy lifestyle [23], being the proposed intervals of ~300 kcal within a 30-min session, this indoor cycling workout could be suggested to reach adequate physical activity levels.

During the recovery period, differences in EE emerged only following DI, probably reflecting the lower intensities administered toward the end of the work-bout when compared to AI and MP. Both DI and RI included the last bout at 50% of the individual PPO. However, in MP it was preceded by the bout at 100% of the individual PPO, thus keeping the intensity higher during the recovery period. Also, it is worth noting that 50% of the PPO approximates the ventilatory threshold and 75% PPO approximates the respiratory compensation threshold [24], thus falling within the heavy exercise intensity domain.

Based on physiological markers, the three sessions showed average values of 72% HR_max_ and 51% VO_2max_, resulting in a moderate intensity exercise [23]. Findings are in line with the study from Battista and colleagues [15] but in contrast with Piacentini et al. [16], in which higher intensity were found (86% HR_max_ and 79% VO_2max_) in an older sample (>30 years of age). sRPE and EES were used to investigate the individual perceptual responses to the exercise protocol. According to sRPE values, subjects rated the indoor cycling sessions as hard (average value = 5) on the Category Ratio 10 scale, corresponding to 14 on the 6–20 RPE scale [18,19], indicating a vigorous intensity exercise [23]. Despite the high intensities perceived, subjects reported “quite a bit” (average value: 5) of enjoyment on the 0–7 EES scale [22], indicating that indoor cycling represents an engaging form of exercise regardless of interval intensity sequencing.

The present study provides useful data regarding the intensity sequencing of indoor cycling, providing evidence that workout structure should be varied to promote adherence and maintain enjoyability, while achieving the same EE. However, some limitations should be acknowledged. Only young recreationally active college students were evaluated, thus future research should be carried out including participants of different ages and activity levels. Moreover, the exercise portion of each session lasted 18 min. As indoor cycling classes in fitness facilities usually last 60 min, future research should focus also on different durations of work bouts.

## 5. Conclusions

The current study supported findings that interval-based exercise routines are time efficient forms of exercise. When considering exercise structure, if total work is kept consistent, the sequence of varying interval bouts has no effect on total exercise session EE, sRPE, or EES. This suggests that group cycling instructors can vary workout structures, alternating different exercise intensities, to promote adherence and maintain enjoyability.

## Figures and Tables

**Table 1:** Mean and standard deviation of the subjects’ characteristics.

	Males (n = 10)	Females (n = 8)	Total (n = 18)
**Age (years)**	20.0 ± 0.8	21.3 ± 2.7	20.6 ± 1.9
**Height (cm)**	180.1 ± 6.7	167.4 ± 3.8	174.4 ± 8.5
**Weight (kg)**	78.4 ± 11.4	66.6 ± 3.6	73.2 ± 10.5
**HR_max_ (bpm)**	193.0 ± 8.0	190.0 ± 7.4	192.0 ± 7.7
**VO_2max_ (mL·kg^−1^·min^−1^)**	47.6 ± 3.7	41.1 ± 4.1	44.7 ± 5.0
**VO_2max_ (L·min^−1^)**	3.7 ± 0.4	2.7 ± 0.2	3.2 ± 0.6
**PPO (W)**	251.0 ± 28.5	201.0 ± 13.9	229.0 ± 34.3

HR_max_: Maximal heart rate; VO_2max_: Maximal oxygen consumption; PPO: Peak power output.

## Data Availability

The data presented in this study are available on request from the corresponding author.
